# Tight Lacing

**Published:** 1871-02

**Authors:** 


Tight Lacing has a great deal to answer for in the pruduction of diseases, by the direct pressure upon the liver, confining it to a smaller space, compressing it, and thus directly preventing its proper action. Lace up an arm or a leg in the same way, and notice how soon the circulation will diminish, the limb decrease in size, and its strength waste away. The effect of continued pressure upon any organ or part of the body is the same. The second way in which it prodnces injury is, by preventing the right mode of breathing. In natural respiration, the diaphragm contracts at every inspiration and forces the liver, stomach and bowels downward and outward, while at each expiration the diaphragm relaxes and the abdominal muscles contract, forcing these organs back to their former position, thus keeping them in constant motion. This motion of respiration is necessary to good digestion, and the healthful action of the liver and bowels. With tight lacing this natural mode of breathing is impossible, and the stomach, liver and bowels being deprived of the needed motion, become torpid and inactive. From inactivity of these organs many of our most dangerous diseases arise.—*Herald of Health.*

